# Clinical-radiomics models based on plain X-rays for prediction of lung metastasis in patients with osteosarcoma

**DOI:** 10.1186/s12880-023-00991-x

**Published:** 2023-03-23

**Authors:** Ping Yin, Junwen Zhong, Ying Liu, Tao Liu, Chao Sun, Xiaoming Liu, Jingjing Cui, Lei Chen, Nan Hong

**Affiliations:** 1 Department of Radiology, Peking University People’s Hospital, 11 Xizhimen Nandajie, Xicheng District, Beijing, 100044 P. R. China; 2 Department of Research and Development, United Imaging Intelligence (Beijing) Co.,Ltd, Yongteng North Road, Haidian District, Beijing, 100089 China

**Keywords:** Osteosarcoma, Radiomics, Lung metastasis, Plain radiographs, Machine learning

## Abstract

**Objectives:**

Osteosarcoma (OS) is the most common primary malignant bone tumor in adolescents. Lung metastasis (LM) occurs in more than half of patients at different stages of the disease course, which is one of the important factors affecting the long-term survival of OS. To develop and validate machine learning radiomics model based on radiographic and clinical features that could predict LM in OS within 3 years.

**Methods:**

486 patients (LM = 200, non-LM = 286) with histologically proven OS were retrospectively analyzed and divided into a training set (n = 389) and a validation set (n = 97). Radiographic features and risk factors (sex, age, tumor location, etc.) associated with LM of patients were evaluated. We built eight clinical-radiomics models (k-nearest neighbor [KNN], logistic regression [LR], support vector machine [SVM], random forest [RF], Decision Tree [DT], Gradient Boosting Decision Tree [GBDT], AdaBoost, and extreme gradient boosting [XGBoost]) and compared their performance. The area under the receiver operating characteristic curve (AUC) and accuracy (ACC) were used to evaluate different models.

**Results:**

The radscore, ALP, and tumor size had significant differences between the LM and non-LM groups (*t*_radscore_ = -5.829, *χ*^*2*^_ALP_ = 97.137, *t*_size_ = -3.437, *P* < 0.01). Multivariable LR analyses showed that ALP was an important indicator for predicting LM of OS (odds ratio [OR] = 7.272, *P* < 0.001). Among the eight models, the SVM-based clinical-radiomics model had the best performance in the validation set (AUC = 0.807, ACC = 0.784).

**Conclusion:**

The clinical-radiomics model had good performance in predicting LM in OS, which would be helpful in clinical decision-making.

**Supplementary Information:**

The online version contains supplementary material available at 10.1186/s12880-023-00991-x.

## Introduction

Osteosarcoma (OS) is the most common primary malignant bone tumor in children and adolescents [1–3]. About 80% of OS occur in the long bones of the extremities, especially in the metaphysis of the distal femur and proximal tibia, and 20% in the axial and pelvic bones [2]. Although the annual incidence of OS is about (1–3) per million people, much lower than common malignant tumors, the disease often has no typical clinical symptoms at the onset, with high malignant degree, great harm and high mortality [2, 4].

Patients with OS are prone to metastasis and have a poor prognosis [5, 6]. Among all the metastases of OS, lung metastasis (LM) are the most common, accounting for more than 70% [7]. The 5-year overall survival and disease-free survival after pulmonary metastasis were 30% and 21%, respectively [8]. Therefore, identifying prognostic factors and timely differentiating patients at high risk can help clinicians develop targeted therapies to improve patients’ outcomes [5]. Previous studies have reported that important indicators of LM in OS patients include age, tumor location, tumor size, chemotherapy, etc. [9–13]. However, no consensus has been reached and these prognostic factors should be further explored and a model for estimating LM in OS patients should be built.

The primary means of OS diagnosis is through evaluating plain X-rays [14]. Early identification of OS by X-ray radiography can reflect tumor size, location, bone destruction, etc. In recent years, some scholars have applied machine learning methods based on X-ray images to the diagnosis and differentiation of OS [14–16]. Alge et al. proposed an automated method for classification of benign tumors and OS using RNA-seq and X-ray images [15]. Hu et al. used image texture analysis to extract texture features from bone X-rays images to evaluate the recognition rate of OS, which can be used in computer-aided OS diagnosis systems [16]. Pereira et al. built machine learning-based computed tomography (CT) radiomic features to predict patients developing metastasis after OS diagnosis [17]. Their model based on Random Forest (RF) achieved an AUC of 0.79 and accuracy of 0.73 of the test set. However, their sample size was relatively small. Plain radiography is routine imaging method for OS in clinical practice, and its radiation dose is lower than CT. Therefore, we hypothesized that X-ray based machine learning models could be used to predict the LM of OS, which would facilitate individualized treatment for patients and increases survival rates.

The objective of this study was to develop and validate clinical-radiomics models based on X-ray features and clinical characteristics to predict the LM of OS patients.

## Materials and methods

### Patients and risk factors

We conducted this retrospective study with the approval of our hospital’s local ethics committee and waived the informed consent requirement. In total, we analyzed data from 550 patients admitted to our hospital from December 2010 to April 2019, all of whom underwent surgery and had pathologically confirmed OS. The inclusion criteria were as follows: (1) X-ray examination was performed within 1 month prior to the initial operation and the tumor was found, (2) X-rays images were of good quality and have no obvious artifacts, and (3) pathologically confirmed OS. Exclusion criteria were as follows: (1) patients lacked preoperative X-rays images, (2) patients had poor image quality, (3) patients lacked of data supporting LM, and (4) patients were lost to follow-up within 3 years. Finally, 486 patients were included and divided into an LM group (n = 200) and a non-LM group (n = 286).

All patients were followed for more than 3 years in this study. Patients were first followed up one month after surgery and monitored every three months for one year after surgery. Each follow-up evaluation included clinical evaluation and imaging evaluation (chest X-ray and/or CT). Patients who presented with clinical symptoms during the unconventional follow-up were also evaluated by X-ray and/or CT. LM was confirmed by biopsy or chest CT images at follow-up. The LM date was determined to be the date when the first CT showed signs of a new lung lesion [18].

**Fig. 1:**
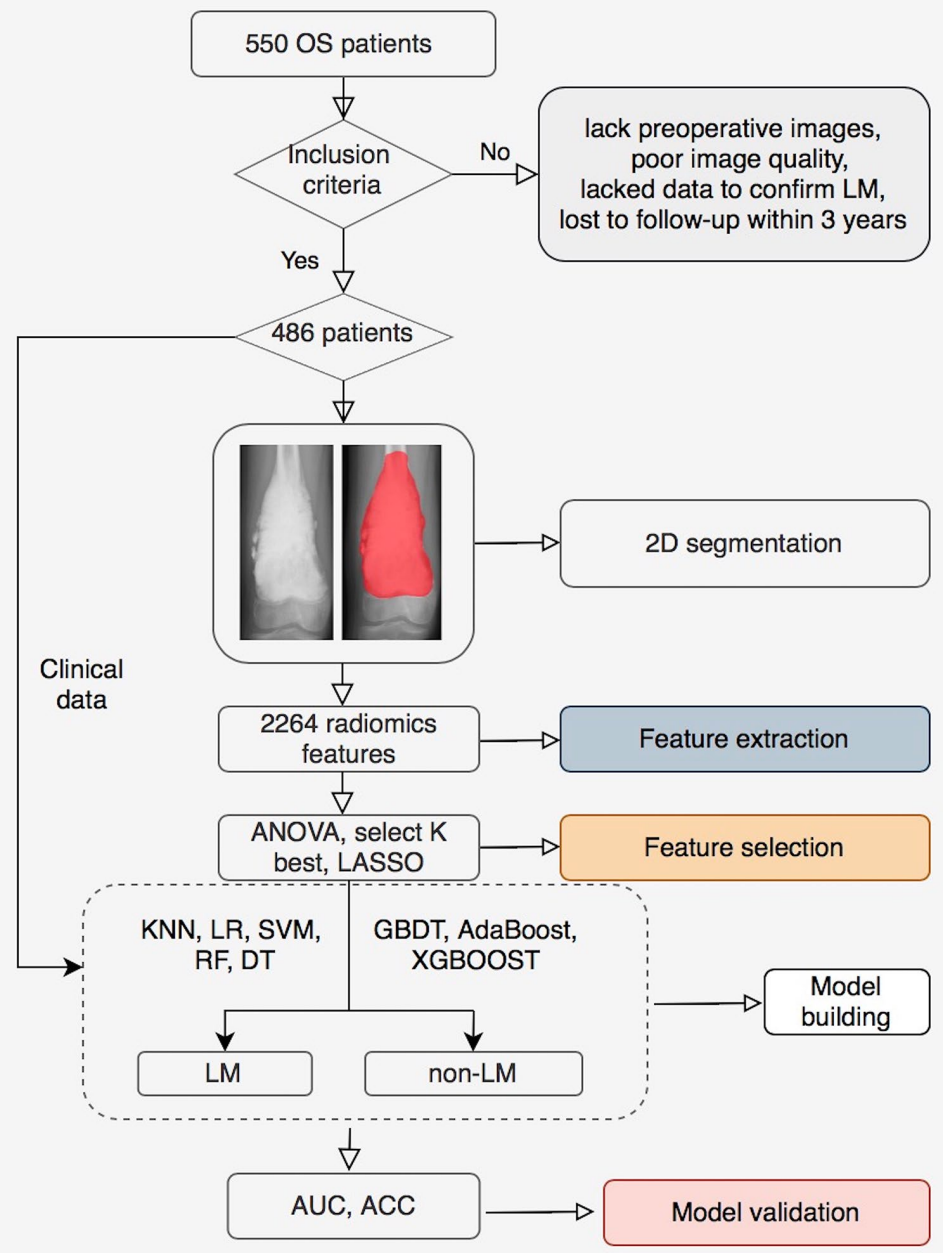
The framework of this study

Risk factors that were potentially associated with LM of OS were analyzed: sex, age, tumor location, maximal tumor size, resection margins, alkaline phosphatase (ALP), and neoadjuvant chemotherapy (NCT). Marginal excision was defined as complete excision of the tumor and its outer membrane, and wide resection was defined as surgical excision beyond the tumor response zone by more than 2 cm. The margins of resection were determined by both surgical records and pathological reports [18]. Figure 1 shows the framework of our study.

### Image acquisition and tumor segmentation

All X-ray digital imaging and communications in medicine images were collected from the picture archiving and communication system of our hospital.

Segmentation of lesions was performed using ITK-SNAP software version 3.6.0 (www.itksnap.org) [19]. All regions of interest (ROIs) were handcrafted along the edge of the tumor on X-ray images that by a musculoskeletal radiologist with 6 years of experience and validated by a senior musculoskeletal radiologist with 15 years of experience.

### Radiomics feature extraction and preprocessing

2264 features were extracted using Research Portal version 1.1 (Shanghai United Imaging Intelligence, Co., Ltd.). Features were divided into three categories, including shape feature, texture feature and gray statistic feature. The above three categories of features were extracted from the original image, and texture feature and gray statistic feature were extracted from the image through a variety of filtering processes. Data preprocessing included resampling the pixel space of X-ray image to 0.2 mm × 0.2 mm, and normalizing the image gray value by max-minimum normalization. The training set and validation set were randomly divided by the ratio of 8:2.

### Radiomics feature screening

To enhance robustness of the radiomic features, the musculoskeletal radiologist draw the ROIs of the compared set (randomly selected 100 patients). We used the intra-class correlation coefficient (ICC) to evaluate inter-reader reproducibility of the features from compared set and original delineation. The features that ICC > 0.75 were retained for later feature selection. Analysis of variance (ANOVA), select K best and least absolute shrinkage and selection operator (LASSO) were used to reduce the redundancy or selection bias of the features. Radiomics score (Radscore) was calculated for each patient via a linear combination of selected features that were weighted by their respective coefficients. λ is the regularization parameter of LASSO regression and is selected when the cross-validation error is minimal [18]. The processing details of the LASSO for feature selection were listed in the Supplementary materials (A).

### Model building and validation

Clinical risk factors were compared via univariate analysis, variables with a *P* value < 0.05 were included in the clinical model. The clinical-radiomics model was established based on the clinical and radiomics characteristics after screening. We built 8 different clinical-radiomics models based on k-nearest neighbor (KNN), RF, support vector machine (SVM), logistic regression (LR), Decision Tree (DT), Gradient Boosting Decision Tree (GBDT), AdaBoost, and extreme gradient boosting (XGBoost) [18, 20]. Finally, we compared the performance of these models. Models were trained using the repeated 5-fold cross-validation method in the training set, and estimation performance was evaluated in the validation set.

The performance of different models was assessed using the area under the receiver operating characteristic curve (AUC) and accuracy (ACC).

### Statistical analyses

R software (R Core Team, Vienna, Austria) version 3.4.3 was used for statistical analysis. The t-test was performed to compare continuous variables, while chi-squared or Fisher’s exact test was used for classify variables between groups. All statistical tests were two-sided, and Bonferroni-corrected *P*-values were used to identify the characteristic significance of multiple comparisons.

## Results

### Clinical characteristics of patients

In total, 486 OS patients (281 males, 205 females; mean age of 19.1 ± 12.8 years, range 3–78 years) were included. Patients in the non-LM group were followed up for more than 36 months, and all the patients remained continually LM-free. In the LM group, the median LM time was 9.5 months (range: 1–36 months). Univariate factor analysis showed that statistically significant differences occurred in radscore, ALP, and tumor size between the two groups (*t*_radscore_ = -5.829, *χ*^*2*^_ALP_ = 97.137, *t*_size_ = -3.437, *P* < 0.01) (Table 1). In the LM group, 123 patients (61.5%) had elevated ALP values, significantly higher than 51 patients (17.8%) in the non-LM group (*χ*^*2*^ = 97.137, *P* < 0.001). In addition, the maximum diameter of tumors in the LM group was significantly larger than that in the non-LM group (*t* = -3.437, *P* = 0.001). No significant difference was found in terms of age, sex, tumor location, resection margins, and NCT between the LM and non-LM group (*t*_*age*_ = -0.328, *χ*^*2*^_*sex*_ = 0.749, *χ*^*2*^_*location*_ = 5.418, *χ*^*2*^_*margin*_ = 0.470, *χ*^*2*^_*NCT*_ = 0.479, *P* > 0.05).

**Table 1:** Clinical characteristics of patients

	LM	non-LM	*χ*^*2*^*/ t* value	*P* value
Sex			*χ*^*2*^ = 0.749	0.387
Male	111 (55.5%)	170 (59.4%)		
Female	89 (44.5%)	116 (40.6%)		
Age (years)				
Mean ± SD	19.4 ± 13.7	19.0 ± 12.2	*t* = -0.328	0.146
Location			*χ*^*2*^ = 5.418	0.367
Femur	123 (61.5%)	165 (57.7%)	*t* = -3.437	0.001
Tibia	35 (17.5%)	70 (24.5%)		
Fibula	7 (3.5%)	9 (3.1%)		
Humerus	22 (11.0%)	21 (7.4%)		
Pelvis	9(4.5%)	12 (4.2%)		
Others	4 (2.0%)	9 (3.1%)		
Size (cm)				
Mean ± SD	10.6 ± 4.6	9.3 ± 4.0		
Surgical resection			*χ*^*2*^ = 0.470	0.493
Wide resection	198 (99.0%)	281 (98.3%)		
Marginal resection	2 (1.0%)	5 (1.7%)		
ALP			*χ*^*2*^ = 97.137	<0.001
Normal	77 (38.5%)	235 (82.2%)		
Elevation	123 (61.5%)	51 (17.8%)		
NCT			*χ*^*2*^ = 0.479	0.489
Yes	163 (81.5%)	226 (79.0%)		
No	37 (18.5%)	60 (21.0%)		

Note: SD = standard deviation, LM = Lung metastasis, ALP = alkaline phosphatase, NCT = neoadjuvant chemotherapy

Multivariable LR analysis showed that ALP was an important independent factor in predicting LM of OS (odds ratio [OR] = 7.272, *P* < 0.001) (Table 2).

**Table 2:** Multivariable logistic regression analyses

Variable	Coefficient	OR (95% CI)	*P*
Intercept	-2.167	0.114	0.039
Age	0.013	1.013 (0.994, 1.033)	0.173
Sex	-0.034	0.967 (0.634, 1.474)	0.875
Radscore	-0.004	0.996 (0.975, 1.018)	0.736
ALP	1.984	7.272 (4.716, 11.215)	< 0.0001
Location	-0.091	0.913 (0.780, 1.069)	0.258
Margin	0.516	1.676 (0.266, 10.555)	0.582
NCT	0.123	1.131 (0.606, 2.113)	0.699
Size	0.039	1.040 (0.989, 1.092)	0.125

Note: OR, odds ratio; CI, confidence interval

### Performance of radiomics models

**Fig. 2:**
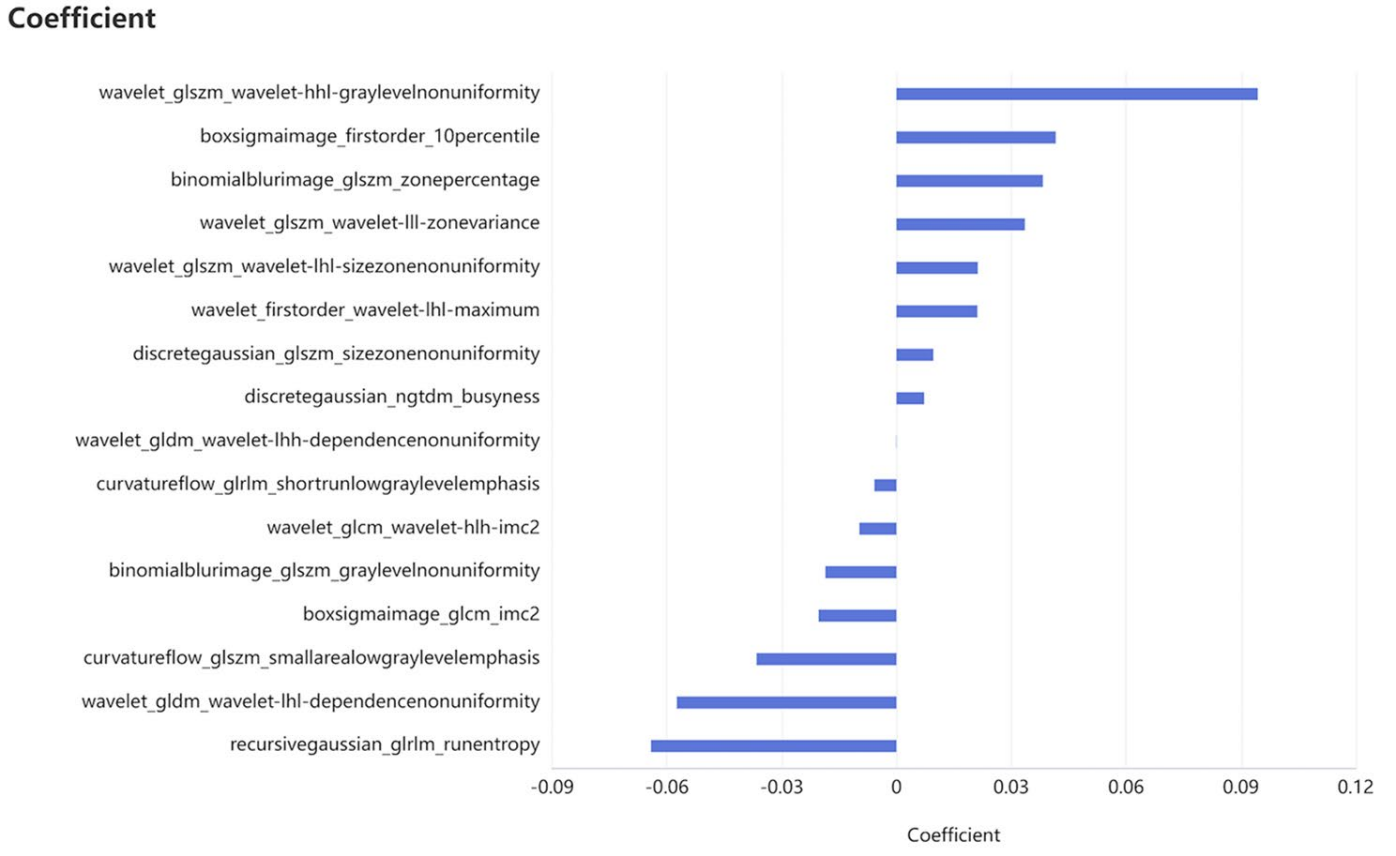
Features were selected to construct the radiomics model

16 radiomics features of each patient were selected for model building, which were shown in the Supplementary materials (B) (Fig. 2).

In the training set, KNN had the highest AUC of 0.864, followed by XGBoost (AUC = 0.862) and AdaBoost (AUC = 0.858), and there was statistical difference in AUC among them (Delong test, *P* < 0.05). As far as ACC was concerned, XGBoost had the highest value of 0.807, followed by Adaboost (0.789) and KNN (0.786).

**Fig. 3:**
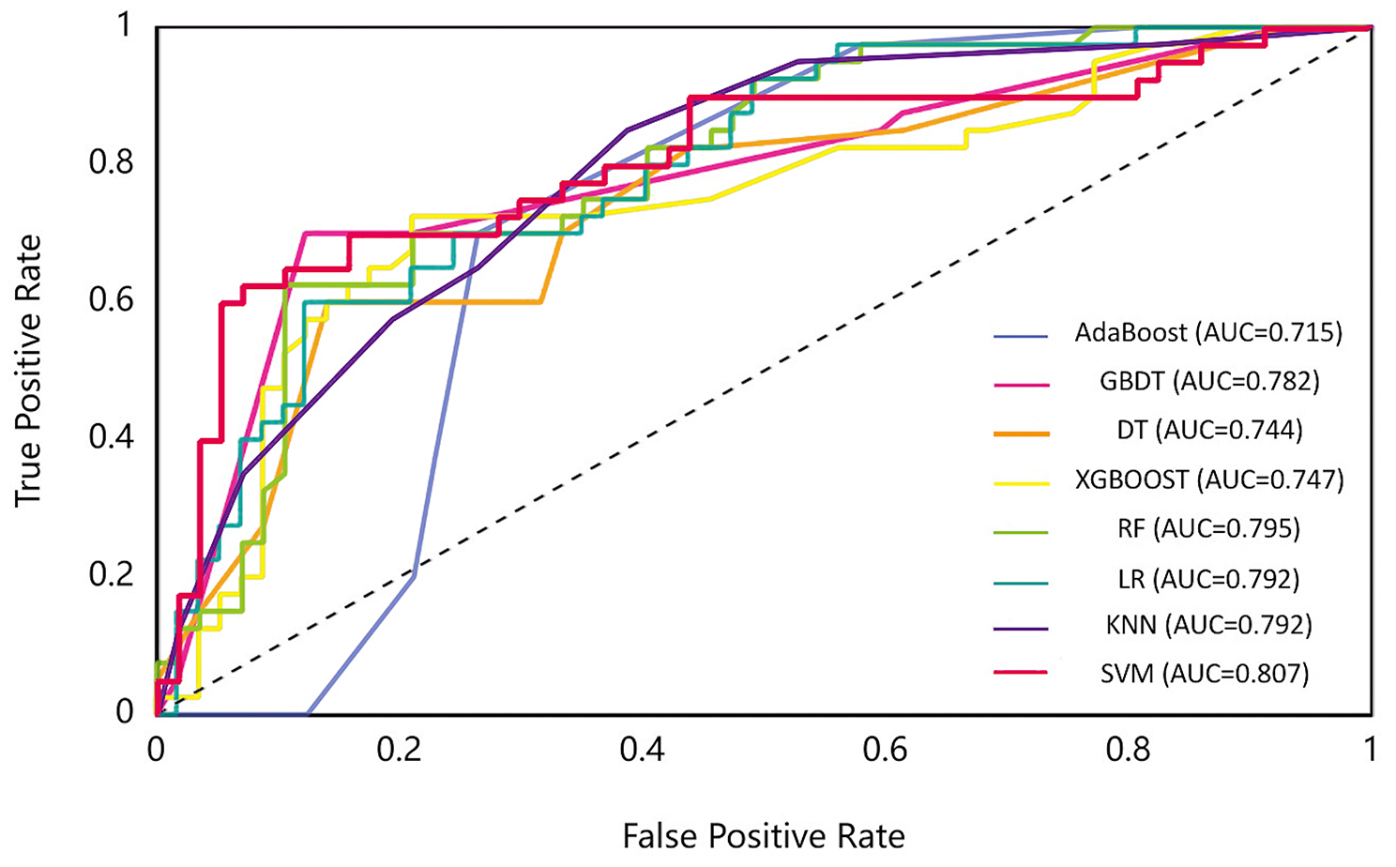
The ROC curve of different models in the validation set. SVM-based clinical-radiomics model achieved the highest performance

In the validation set, SVM had the highest AUC of 0.807, followed by RF (AUC = 0.795), KNN (AUC = 0.792), LR (AUC = 0.792), GBDT (AUC = 0.782), XGBoost (AUC = 0.747), DT (AUC = 0.744), and AdaBoost (AUC = 0.715). In terms of ACC, GBDT had the highest value (0.794), followed by SVM (0.784), Adaboost (0.753), and XGBoost (0.753). The ACC of SVM was slightly lower than that of GBDT, but the AUC of SVM was significantly higher than that of GBDT (Delong test, *P* < 0.05). Although the AUC of SVM was slightly higher than that of AdaBoost without statistical difference (Delong test, *P* > 0.05), ACC value of SVM was also higher than that of AdaBoost. In addition, the AUC of SVM is significantly higher than RF, LR, KNN and XGBoost, with statistical difference (Delong test, *P* < 0.05). Therefore, considering AUC and ACC, SVM-based clinical-radiomics model performed better (AUC = 0.807, ACC = 0.784, sensitivity = 0.700, specificity = 0.842) (Fig. 3; Table 3).

**Table 3:** Performance of radiomics models in the validation set

	AUC	ACC	Sensitivity	Specificity	F1score	Precision
KNN	0.792	0.701	0.650	0.737	0.642	0.634
LR SVM RF	0.792 0.807 0.795	0.742 0.784 0.732	0.600 0.700 0.700	0.842 0.842 0.754	0.658 0.727 0.683	0.727 0.757 0.667
DT	0.744	0.649	0.600	0.684	0.585	0.571
GBDT	0.782	0.794	0.700	0.860	0.737	0.778
AdaBoost	0.715	0.753	0.750	0.754	0.714	0.682
XGBoost	0.747	0.753	0.650	0.825	0.684	0.722

Note: AUC = area under curve, ACC = accuracy, LR = logistic regression, RF = random forest, DT = Decision Tree

## Discussion

In this study, we found that statistically significant differences in radscore, ALP, and tumor size between the LM and non-LM groups. However, multivariable LR analysis showed that ALP was an important independent factor in predicting LM in OS patients. SVM-based clinical-radiomics model reached an AUC of 0.807 and ACC of 0.784 in the validation set, which was higher than other models.

Univariate factor analysis showed that statistically significant differences occurred in radscore, ALP, and tumor size between the two groups. Furthermore, ALP was an important independent predictor of LM in OS patients. ALP is a frequently used clinical indicator for the diagnosis of OS, and the National Comprehensive Cancer Network guidelines show that ALP is associated with the diagnosis and prognosis of OS [21, 22]. Wang et al. [23] found that ALP was associated with 5-year LM-free survival of OS patients. Luo et al.[13] found that ALP was not significant clinical indicator for prediction of synchronous LM. Contrary to their results, our study showed that ALP was an independent predictor of LM in OS patients. Radscore can reflect the heterogeneity of different tumors, which has been widely used [24, 25]. Yin et al. [25] found that radscore was important predictor for differentiating benign and malignant sacral tumors. In addition, we found that the maximum size of tumors in the LM group was larger than that in the non-LM group. However, our study showed that tumor size was not an independent factor in predicting LM, which was inconsistent with previous studies [13, 26]. Huang et al. [10] reported that OS patients with large tumors (> 5 cm) have a significantly increased risk of pulmonary metastasis. In addition, we found no significant differences in age, sex, tumor location, resection margin, and NCT between the LM group and the non-LM group, which is inconsistent with some previous studies [5, 10, 27]. This may be related to the fact that our study took 3 years as a node and the sample size was relatively small.

In this study, we compared eight frequently used machine learning methods in predicting the occurrence of LM in OS patients, and these algorithms also performed well in previous studies [13, 17, 20, 25]. Our results demonstrated that KNN performed best in the training group. KNN is a conceptually simple but powerful algorithm that is easy to use, interpret and implement [28]. It has been widely used in previous studies [29–31]. Furthermore, we found that XGBoost had the highest ACC in the training group. XGBoost is a new large-scale machine learning algorithm that minimizes the loss of functions through iterative construction [32, 33]. Although we found that XGBoost had a relatively low AUC of 0.747 in the validation group, it was higher than the results of previous study [34]. Therefore, we still believe it is a good machine learning method for predicting LM in OS patients. In the validation set, we found that SVM-based clinical-radiomics model exhibited a better performance than other models. SVM has been widely used because it can solve small sample, nonlinear problems and has strong generalization ability [13, 20]. It has been used to predict the efficacy of neoadjuvant chemotherapy [4], synchronous LM [13], and overall survival [35] in OS patients. RF also performed well in the validation set, second only to SVM. RF is a very effective model-free classification method, which is robust to noise and outliers, and can deal with high dimensional space quickly, but there are overfitting problems [20]. As far as ACC is concerned, GBDT performed best in the validation set. The GBDT model has the advantages of flexibility, high predictive power, and effectiveness in processing imbalanced data [36]. Our SVM-based clinical-radiomics model can help doctors determine the risk of LM in OS patients and make personalized medical plans in time. For high-risk patients, doctors may recommend closer monitoring, increased chemotherapy, or more sensitive chemotherapy regimens [23].

Our study has certain limitations. First, all the images were acquired over the course of several years from one center. We excluded some patients who did not receive X-ray before surgery (e.g., some patients have already been examined in other hospitals), which may lead to selection bias. Although we conducted strict screening and preprocessing of the included data, a multicenter study with a larger sample size is beneficial for further study. Second, we only compare 8 commonly used classifiers, and the performance of other machine learning methods or deep learning algorithms needs to be further studied. Third, although plain radiography is the routine preferred examination and prevention for patients, the information provided is still limited. In the future, building a multi-modal combined model combined with CT and MRI may provide more information for patients’ prognosis.

In conclusion, the clinical-radiomics model had good performance in estimating LM of OS patients, especially based on SVM algorithm, which would be helpful in clinical decision-making.

## Electronic supplementary material

Below is the link to the electronic supplementary material.

Supplementary Material 1

## Data Availability

The datasets generated during and/or analysed during the current study are available from the corresponding author on reasonable request.

## Abbreviations

OS: Osteosarcoma
LM: lung metastasis
RF: Random Forest
ALP: alkaline phosphatase
ROIs: Regions of interest
ANOVA: Analysis of variance
LASSO: Least absolute shrinkage and selection operator
Radscore: Radiomics score
KNN: k-nearest neighbor
SVM: support vector machine
LR: logistic regression
DT: Decision Tree
GBDT: Gradient Boosting Decision Tree
XGBoost: extreme gradient boosting
AUC: Area under the curve
ACC: Accuracy.
