# Supplementary material for: Clinical-radiomics models based on plain X-rays for prediction of lung metastasis in patients with osteosarcoma
Source: BMC Med Imaging. 2023 Mar 23;23:40. doi: 10.1186/s12880-023-00991-x (PMC10037898; doi:10.1186/s12880-023-00991-x)
Supplement: Supplementary file 1 — Supplementary Material 1 [file 12880_2023_991_MOESM1_ESM.doc]

**A:** **The processing details of the LASSO for feature selection.**

In this study, the least absolute shrinkage and selection operator (LASSO) logistic regression algorithm was performed to reduce the high-dimensional features. LASSO regression eliminated the coefficients for lung metastasis-unrelated variables to zero and contained the variables with non-zero coefficients. The optimal parameter (Alpha=0.009, -Log(Alpha)=2.05) was selected by 5-fold cross-validation. The non-zero coefficients of the maintained features at optimal parameter are shown in Supplementary Figure 1.


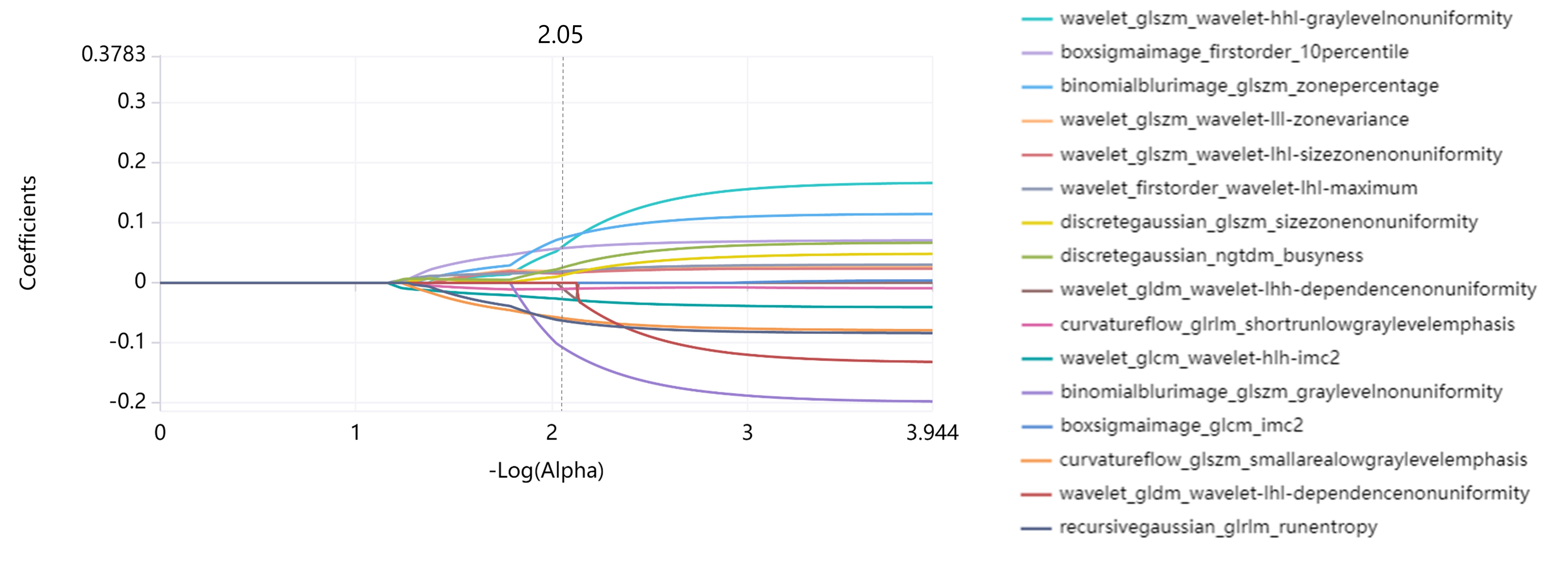


Supplementary Figure 1

Then, a radiomics score (radscore) was generated by a linear combination of selected features multiplied by corresponding LASSO coefficients. The calculation formula is:

Radscore = wavelet_glszm_wavelet-hhl-graylevelnonuniformity×0.09439616

+ boxsigmaimage_firstorder_10percentile×0.04161222

+binomialblurimage_glszm_zonepercentage×0.0382517949

+wavelet_glszm_wavelet-lll-zonevariance×0.033543136

+wavelet_glszm_wavelet-lhl-sizezonenonuniformity×0.02124879

+wavelet_firstorder_wavelet-lhl-maximum×0.0211289469

+discretegaussian_glszm_sizezonenonuniformity×0.009631921

+discretegaussian_ngtdm_busyness×0.0072335843

+wavelet_gldm_wavelet-lhh-dependencenonuniformity×-0.0000825031

+curvatureflow_glrlm_shortrunlowgraylevelemphasis×-0.005743311

+wavelet_glcm_wavelet-hlh-imc2×-0.009685197

+binomialblurimage_glszm_graylevelnonuniformity×-0.0185575783

+boxsigmaimage_glcm_imc2×-0.0203487985

+curvatureflow_glszm_smallarealowgraylevelemphasis×-0.0365530849

+wavelet_gldm_wavelet-lhl-dependencenonuniformity×-0.05738717

+recursivegaussian_glrlm_runentropy×-0.06413293

**B: 16 radiomics features.**

16 radiomics features of each patient were selected for model building, including wavelet_glszm_wavelet-hhl-graylevelnonuniformity, boxsigmaimage_firstorder_10percentile, binomialblurimage_glszm_zonepercentage, wavelet_glszm_wavelet-lll-zonevariance, wavelet_glszm_wavelet-lhl-sizezonenonuniformity, wavelet_firstorder_wavelet-lhl-maximum, discretegaussian_glszm_ sizezonenonuniformity, discretegaussian_ngtdm_busyness, wavelet_gldm_wavelet-lhh-dependencenonuniformity, curvatureflow_glrlm_shortrunlowgraylevelemphasis, wavelet_glcm_wavelet-hlh-imc2, binomialblurimage_glszm_graylevelnonuniformity, boxsigmaimage_glcm_imc2, curvatureflow_glszm_smallarealowgraylevelemphasis, wavelet_gldm_wavelet-lhl- dependencenonuniformity, recursivegaussian_glrlm_runentropy.
